# Longitudinal trajectories of the overall and regional body composition between severe acute malnourished and well-nourished children of Rohingya refugee camps

**DOI:** 10.3389/fpubh.2024.1442142

**Published:** 2024-10-31

**Authors:** Mohammad Zahidul Manir, A. K. Obidul Huq

**Affiliations:** ^1^Department of Food Technology and Nutritional Science, Mawlana Bhashani Science and Technology University, Santosh, Tangail, Bangladesh; ^2^UNICEF, Dhaka, Bangladesh

**Keywords:** body composition, fat mass, fat-free mass, severe acute malnutrition, children under 5 years of age, Rohingya refugee, longitudinal study

## Abstract

**Objectives:**

The present study aimed to observe how body composition differs between severe acute malnutrition (SAM) (treated with ready-to-use therapeutic food, RUTF) and well-nourished children.

**Methods:**

A longitudinal investigation was conducted among well-nourished and SAM children of 6-59 months in Rohingya refugee camps. These two groups (350 children in each group) of children were observed over 12 weeks and individual data were collected during admission, follow-up visits, and at the time of discharge. Anthropometric information was collected following standard procedures. The thicknesses of the biceps, triceps, subscapular, and supra iliac skinfolds were measured using a Herpenden-type skinfold caliper. Separate linear mixed models were conducted to assess associations of the independent variables (i.e., group and time) with each of the dependent variables (i.e., biceps, triceps, subscapular, supra-iliac skinfold thickness (ST), fat mass (FM), and fat-free mass (FFM)).

**Results:**

Both in well-nourished and SAM children, the mean biceps, triceps, subscapular, and supra-iliac ST, FM, and FFM increased over the 12 weeks. The increase in biceps ST was significantly faster in the SAM children compared to the well-nourished children (difference in slope = 0.366 mm every four weeks; *p* < 0.001). The increment rate in triceps ST was also faster in the SAM children compared to the well-nourished children (difference in slope = 0.430 mm every four weeks; *p* < 0.001). Moreover, the pace of increase in subscapular (difference in slope = 0.027 mm every four weeks; *p* < 0.001), and supra-iliac (difference in slope = 0.211 mm every four weeks, *p* < 0.001) ST was also significantly higher in the SAM group. Similarly, the change in FM (difference in slope = 0.065 kg every four weeks, *p* < 0.001) and FFM (difference in slope = 0.152 kg every four weeks, *p* = 0.023) was also significantly faster in SAM children compared to the well-nourished children over the treatment period. Furthermore, the girls gained significantly higher triceps ST, subscapular ST, FM, and FFM compared to the boys.

**Conclusion:**

The benefit of RUTF was evident from this longitudinal study in the recovery of FM and FFM contents among the SAM children of Rohingya refugee camps.

## 1 Introduction

Approximately half of the deaths in children under five are attributed to undernutrition, despite significant advancements in its prevention and treatment (1). Thus, child undernutrition remains a serious global health concern. Moreover, a gap exists regarding information about how undernutrition affects different aspects of body composition and function. Fat-free mass (FFM) and fat mass (FM) are the two components of mass in the most fundamental body composition model, which provides insights into the physical consequences of undernutrition (2).

Previous studies have shown that both fat and fat-free tissues are affected in a wasted child (3). Although the distribution of fat appears to be more central, wasting is more closely linked to the decrease of peripheral fat than to the increase of central fat. Moreover, stunting (chronic undernutrition) is linked to deficiencies in FFM and other aspects, such as organ size. This implies that rather than peripheral growth, FFM is primarily reduced in proportion to linear expansion. The degree of FM in stunted children varies; some of them may remain thin throughout infancy, while others may experience increased degrees of FM. The causes of this heterogeneity are still unknown. The longitudinal relationships between early stunting and later body composition may be explained through multiple routes. Notably, new research indicates that malnourished children who receive nutritional supplements may not be at risk for short-term excess fat deposition (3).

The Rohingya people are one of the most persecuted population groups in the world. Nearly 0.7 million people fled from Myanmar to Bangladesh in the second half of 2017 to escape rape, forced labor, land expropriation, death, etc. by the military regime in Myanmar (4, 5). Since 1992, there have been 0.25 million pre-resided refugees. This new influx persisted until 2018, when 1.2 million Rohingya refugees were living in Cox’s Bazar’s registered camps (RCs) and makeshift settlements (MSs), causing an extreme humanitarian crisis of sanitation, health, and nutrition (6). During April to May 2018, wasting (acute malnutrition) had significantly decreased and remained below emergency levels, a year after the acute phase of the emergency. A comparable pattern in wasting was noted when measured using low weight-for-height z-score (WHZ) or low mid-upper arm circumference (MUAC) (7). Although the level of wasting improved significantly compared to the prevalence measured at the beginning of the influx, it still remains above the WHO global threshold. The United Nations High Commissioner for Refugees (UNHCR) Bangladesh 2023 Standardized Expanded Nutrition Survey (SENS) revealed that the acute malnutrition situation in the Rohingya Forcibly Displaced Myanmar Nationals (FDMN) camps was deteriorating and presented a very high public health concern (15.4%), while the situation in the registered camps remained unchanged and posed a medium public health concern (9.6%) (8). Sustained efforts to identify and treat children at the onset of malnutrition are necessary for further progress. It seems that by rectifying tissue deficiencies and their functional impacts, evidence-based knowledge in this area may facilitate the advancement of clinical and public health interventions, which in turn may enhance long-term quality of life and survival.

However, there has previously been evidence of discrepancies in the identification of child acute malnutrition using WHZ and MUAC scores in Bangladesh, Myanmar, and other parts of the world (5, 9). Therefore, in the camps, both WHZ and MUAC are used for the diagnosis, admission, and discharge of children with severe acute malnutrition (SAM). This phenomenon indicates the importance of body composition in the nutritional status of the children. In the Rohingya FDMN camps, children with SAM are being treated with ready-to-use therapeutic food (RUTF). RUTF is a highly nutritious, energy-dense food made from ingredients such as peanuts, vegetable oil, powdered milk, butter, sugar, and vital micronutrients. Each RUTF sachet contains 500 calories along with micronutrients, providing malnourished children with the necessary nutrients to gain weight rapidly. Very little literature exists on the differences in body composition among children who receive RUTF as a nutritional treatment. Thus, this study was designed to observe how body composition differs between SAM and well-nourished children. This could add value to global literature by enhancing the understanding of undernutrition and by helping to design better treatment strategies.

## 2 Methods and materials

### 2.1 Study design and setting

The present longitudinal investigation was conducted among well-nourished children and children with SAM of 6–59 months in Rohingya refugee camps. Individual data were collected during admission, follow-up visits, and discharge. The study was conducted in the FDMN camp in Ukhiya, Cox’s Bazar, which includes several areas, including Camp 8E, Camp 9, Camp 11, and Camp 18.

### 2.2 Sample size and sampling technique

Children aged between 6 and 59 months (both malnourished and well-nourished) residing in the FDMN camps in Ukhiya, Cox’s Bazar, were selected as participants in the present study. Two groups of children (SAM and well-nourished) were included in this longitudinal study and were observed over 12 weeks. A total of 350 children were included in each group.

The sample size was calculated using the following Cochrane formula: *n* = Z^2^pq/d^2^. For the SAM group, the parameters were as follows: p = the prevalence of the children with SAM in the Rohingya camp population (e.g., SAM prevalence by WHZ and/or MUAC is 0.9%, SMART Survey 2019), q = 1-p, d = margin of error (1%), Z = 1.96 taken at a 95% confidence interval, and a 2% non-response rate (loss of cases, default, refusal, incompleteness, etc.). For the well-nourished group, the parameters were as follows: p = the prevalence of the well-nourished children (prevalence of well-nourished by WHZ and/or MUAC is 87.8%, SMART survey 2019), q = 1-p, d = desired precision (3.5%), Z = 1.96 taken at a 95% confidence interval, and a 4% non-response rate (loss of cases, default, refusal, incompleteness, etc.).

UNICEF provides nutritional support to children with SAM through 26 integrated nutrition facilities (INFs) managed by its three implementing partners. The study focused on camps located in areas such as Camp 8E, Camp 9, Camp 11, and Camp 18, which are part of a larger network of camps for Rohingya refugees. A total of four INFs were selected to enroll the desired number of children from Upazila Ukhia. To minimize selection bias, the INFs were randomly selected from all three implementing partners. For the selection of cured and non-cured cases, all children from the selected INFs were enrolled in this study to meet the estimated number of cases among children with SAM who were admitted to the nutrition centers. For the selection of well-nourished cases, a systematic random sampling technique was used to meet the estimated number of samples among children who came to the INFs for growth monitoring and promotion.

### 2.3 Inclusion and exclusion criteria

Children aged 6–59 months residing in the FDMN and mothers who provided consent for collecting data were included in this study. The exclusion criteria were as follows: (a) children outside the age range of 6–59 months; (b) those who were not residing in the FDMN; (c) incomplete data; (d) those with existing medical conditions such as renal disease or severe abnormalities; and (e) those who were not interested in participating.

### 2.4 Recruitment process and RUTF dose administration

The study included children aged 6–59 months, both malnourished and well-nourished, residing in the FDMN camps in Ukhiya, Cox’s Bazar. Community nutrition volunteers screened children using MUAC measurements at the outreach level. The children with a MUAC measurement below 135 mm were referred to the INFs for a full assessment, while those identified with SAM based on MUAC or weight-for-height (WHZ) were considered eligible for the study according to the inclusion criteria. SAM was defined as a WHZ value less than −3 or a MUAC value less than 115 mm and/or the presence of edema.

For the treatment of SAM, RUTF is administered at a particular dose, frequency, and for a certain period. The daily dosage of RUTF is calculated based on the child’s weight. The study followed the standard recommendation of 200 kcal per kilogram of body weight per day. RUTF was provided in two to three meals per day, depending on the child’s needs and the prescribed amount. The nutrition worker at the INFs provided RUTF for 7 days as a take-home ration. The treatment was continued until the child reached the target weight and was no longer classified as severely malnourished, which typically takes 6–8 weeks and a maximum length of stay (LOS) of 12 weeks.

### 2.5 Data collection

During admission, data on anthropometric measurements and body composition were collected as baseline, along with demographic data (e.g., gender, parent’s education, occupation, income status, age of the mother, and source of income.). A pre-tested questionnaire was used for the collection of background data. Subsequently, only anthropometric and body composition-related data were collected during each follow-up visit until the children were discharged.

### 2.6 Anthropometric assessment

Standard procedures were followed to measure the anthropometric information (weight, height, MUAC, and edema) (10). Z-scores were calculated using WHO Anthro software according to the 2006 WHO growth standards (11). SAM was defined as a WHZ value < −3, a MUAC value <115 mm, and/or the presence of edema. A weighing scale was used to estimate the weight of the children. Each child was measured twice. A third measurement was taken if there was a difference of more than 100 g between the first two measurements. The average of the closest two measurements was recorded as the final weight of the children.

For the children aged from 6 months to 2 years, length was measured by placing the children straight on an infantometer. For the children over 2 years, height was measured using a stadiometer, with each child standing with their back and shoulders erect. Each child was measured twice. If the differences between the height/length measurements were > 0.7 cm, a third measurement was considered. The children’s final length and height were calculated as the average of the closest measurements. A non-stretchable fiberglass MUAC tape was used to collect the MUAC measurements of each child.

### 2.7 Measurement of the body composition

The thicknesses of the biceps, triceps, subscapular, and suprailiac skinfolds were measured on the left to the nearest 0.2 mm using a Herpenden-type skinfold caliper. The subscapular skinfold was identified as the inferior angle of the scapula, at a 45° angle parallel to the interior angle and 2 cm below the scapula. The suprailiac skinfold thickness (ST) was measured 1–2 cm above the wing of the ilium and at an approximate 45° angle from the body. The triceps skinfold was identified as a vertical fold on the triceps, located at mid-height. The biceps skinfold was identified as a vertical fold on the biceps, located at the mid-height of the arm. A standard methodology was followed by skilled observers (research nurses and dieticians) to conduct the measurements. Each measurement was estimated after the caliper pointer was calibrated.

The following formula was used to estimate the body fat percentage of the children using the four STs (12).


$$\begin{array}{l} \mathrm{Body}\;\mathrm{fat}\;\left(BF\right)\%=8.71+\left(0.19\times \mathrm{subscapular in}\;\mathrm{mm}\right)\\ \;+\left(0.76\times \mathrm{biceps in}\;\mathrm{mm}\right)\\ \;+\left(0.18\times \mathrm{suprailiac in}\;\mathrm{mm}\right)\\ \;+\left(0.33\times \mathrm{triceps in}\;\mathrm{mm}\right) \end{array}$$

FM=%BF×weight

$$FFM=\left(\mathrm{weight}–FM\right)$$

### 2.8 Ethical issue

Before proceeding with the study, the proposal was submitted to the ethics review committee of the Department of Food Technology and Nutrition Science of Mawlana Bhashani Science and Technology University for approval. UNICEF-supported nutrition programs require approval from national-and local-level nutrition, health, and administrative authorities. Thus, administrative approval was obtained from the Refugee Relief and Repatriation Commissioner (RRRC) and UNICEF before data collection. Written consent was obtained from the participants and recorded regarding consent for publication.

### 2.9 Statistical analysis

For categorical variables, descriptive statistics were expressed as frequency (n, %). A chi-squared (χ2) test was used to compare the sociodemographic characteristics of the children among the well-nourished and SAM groups. To assess associations between the independent variables (group: well-nourished and SAM), time, and the dependent variables (biceps, triceps, subscapular, suprailiac ST, FM, and FFM), separate linear mixed models were conducted. The following prespecified covariates were included in the models: the children as random (intercept) effects, the intervention groups as fixed effects, time (follow-up visits), and the interaction between the group and time. Moreover, to evaluate the comparative changes in the body composition of the boys and girls of the two groups, separate linear mixed-effects models were conducted. The gender of the children, their group, and the interaction of the gender and group were included as fixed effects. The children were included as random (intercept) effects. Relevant findings were then presented as percentage changes from baseline values, along with matching 95% confidence intervals. To verify the model assumptions for each analysis, residual and normal probability plots were employed. An independent sample *t*-test was conducted to assess the significant difference between the groups at each time point. *p*-values were considered significant if they were less than 0.05. For all the statistical analyses, SPSS software (version 25.0) was used.

## 3 Results

### 3.1 Sociodemographic characteristics of the children

The boys comprised a larger proportion of the well-nourished children (55.1% vs. 44.9%), while the girls comprised a larger proportion of the SAM group (59.4% vs. 40.6%) (Table 1). In the SAM group, only 13.7% of the children were 24–59 months old, while over half (53.4%) were 6–11 months old. The majority of the mothers in the SAM and well-nourished groups had completed at least primary school. While only 22.3% of the children who were well-nourished were observed to consume water from other sources without further purification, a greater proportion of the children with SAM (41.1%) were observed to consume water from other sources with further purification. (Table 1).

**Table 1:** Sociodemographic characteristics of the severe acute malnourished and well-nourished children aged 6 to 59 months in the Rohingya camp.

Sociodemographic characteristics	Well-nourished children n (%)	SAM children n (%)	*p*-value
Age of the children (months)			
6–11	98 (28.0)	187 (53.4)	
12–23	146 (41.7)	115 (32.9)	<0.001
24–59	106 (30.3)	48 (13.7)	
Gender of the children			
Boy	193 (51.1)	142 (40.6)	<0.001
Girl	157 (44.9)	208 (59.4)	
Residence area			
Camp 8E	66 (18.9)	94 (26.9)	
Camp-9	145 (41.4)	54 (15.4)	
Camp-11	68 (19.4)	116 (33.1)	<0.001
Camp-18	71 (20.3)	86 (24.6)	
Household income			
Yes	139 (39.7)	151 (43.1)	0.399
No	211 (60.3)	199 (56.9)	
Mother’s education			
No formal education	39 (11.1)	19 (5.4)	0.009
Having at least primary education	311 (88.9)	331 (94.6)	
Source of drinking water			
Tubewell water	257 (73.4)	193 (55.1)	
Boiled water	15 (4.3)	13 (3.7)	<0.001
Direct water from other sources	78 (22.3)	144 (41.1)	
Handwashing status			
Yes	338 (96.4)	303 (86.6)	<0.001
No	12 (3.4)	47 (13.4)	
Sanitary latrine facility			
Yes	243 (69.4)	176 (50.3)	<0.001
No	107 (30.6)	174 (49.7)	

SAM, Severe acute malnourished, *p*-value from chi-squared test.

### 3.2 Body composition of the children

**Figure 1:**
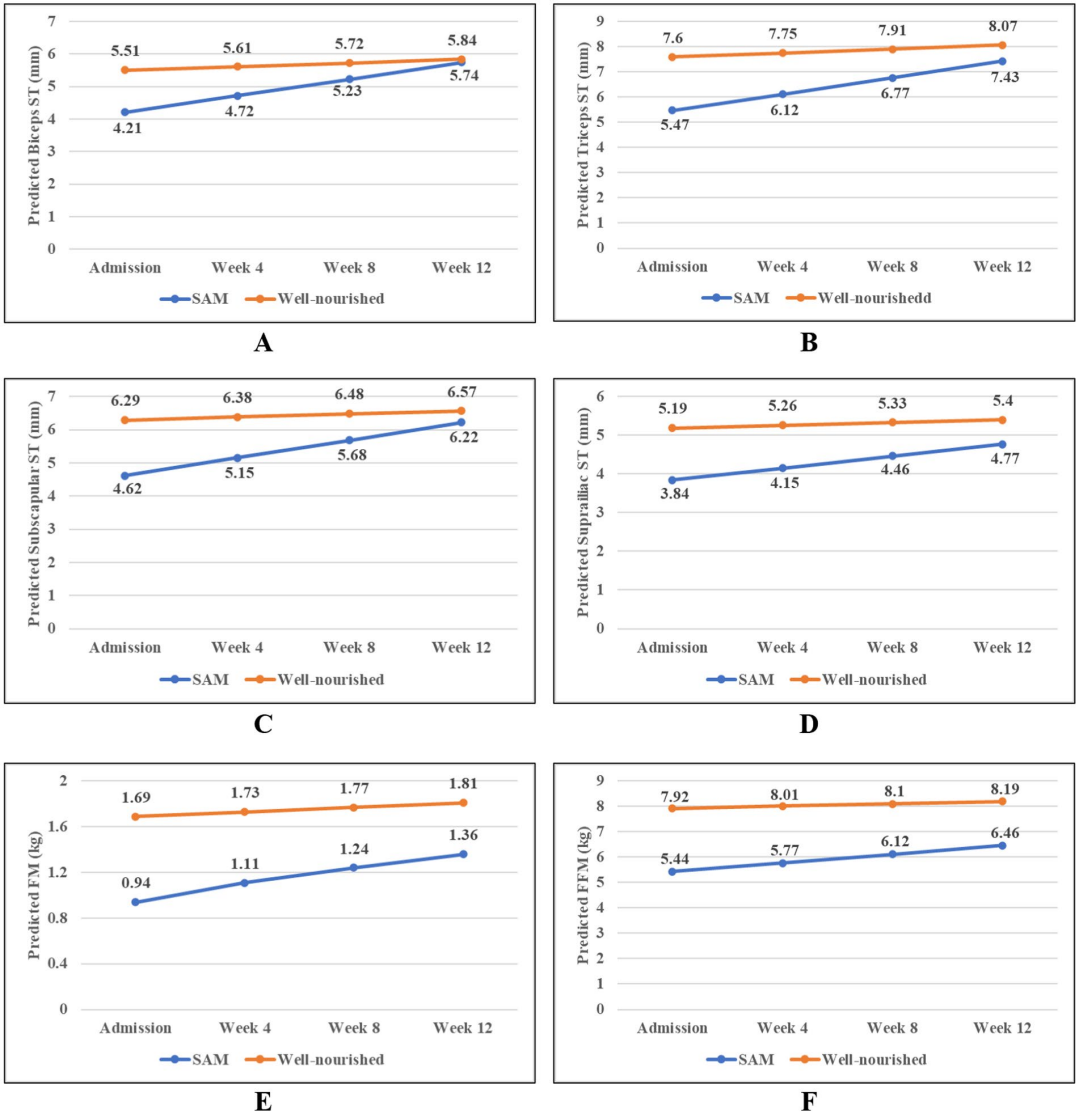
Longitudinal trajectories in the overall and regional body composition among the children with severe acute malnutrition (SAM) and the well-nourished children; (A) Biceps, (B) Triceps, (C) Subscapular, (D) Suprailiac, (E) Fat mass, and (F) Fat-free mass. Predicted values of the body composition parameters were obtained from the linear mixed model.

Figure 1 shows the longitudinal trajectories of the overall and regional body composition among the severe acute malnourished and well-nourished children. The mean thickness of the biceps, triceps, subscapular, and suprailiac fat increased as time increased from baseline in both well-nourished children and children with SAM. However, the increase was higher among the children with SAM compared to the well-nourished children. Similarly, the mean FM and FFM increased in both groups, with a sharp increase observed in the children with SAM. After 12 weeks, there was no significant difference in the biceps ST between the SAM and well-nourished groups. However, for the triceps, subscapular, and suprailiac ST, the differences between the two groups were statistically significant at each time point, although the gap between the two groups reduced over time (Supplementary Table S1). A similar pattern was found for the FM and FFM. The changes in weight and MUAC between these groups also showed a higher rate of increase in the SAM group compared to the well-nourished group (Supplementary Figure S1). Similarly, when the changes in the FM and FFM were observed between these two groups, considering different age stages, a higher rate of increase in the FM and FFM was found in the SAM group compared to the well-nourished group (Supplementary Figure S2).

### 3.3 Comparative changes in the body composition of the well-nourished children and the children with SAM

Both in well-nourished and children with SAM, the mean thickness of the regional fat (biceps, triceps, subscapular, and suprailiac), overall FM, and FFM increased over the 12 weeks (Table 2). The rate of change in the biceps, triceps, subscapular, suprailiac, FM, and FFM was higher in the children with SAM than in the well-nourished children. However, the rate of change decreased over time. During the 12 weeks of treatment, the increase in the biceps ST was significantly faster in the children with children compared to the well-nourished children (difference in slope = 0.366 mm every 4 weeks; *p* < 0.001; Table 2).

**Table 2:** Comparative changes in the body composition of the well-nourished children and the children with SAM.

Body composition	β (95% CI)	t	*p*-value
Biceps			
Intercept	6.342 (5.878, 6.806)	26.814	<0.001
Group	0.196 (−0.459, 0.067)	−1.464	0.113
Time	−0.844 (−0.998, −0.690)	−10.759	<0.001
Group*Time	0.366 (0.277, 0.455)	8.059	<0.001
Triceps			
Intercept	7.499 (6.914, 8.084)	25.142	<0.001
Group	0.363 (0.032, 0.695)	2.151	0.032
Time	−1.017 (−1.211, −0.823)	−10.287	<0.001
Group*Time	0.430 (0.318, 0.542)	7.5	<0.001
Subscapular			
Intercept	6.615 (6.159, 7.072)	28.401	<0.001
Group	0.027 (−0.232, 0.285)	0.201	0.841
Time	−0.903 (−1.055, −0.752)	−11.694	<0.001
Group*Time	0.404 (0.316, 0.492)	9.032	<0.001
Suprailiac			
Intercept	4.485 (4.036, 4.933)	19.598	<0.001
Group	0.491 (0.236, 0.745)	3.785	<0.001
Time	−0.490 (−0.638, −0.341)	−6.454	<0.001
Group*Time	0.211 (0.124, 0.297)	4.791	<0.001
FM			
Intercept	0.983 (0.820, 1.145)	11.866	<0.001
Group	0.432 (0.340, 0.524)	9.217	<0.001
Time	−0.170 (−0.224, −0.116)	−6.185	<0.001
Group*Time	0.065 (0.034, 0.097)	4.104	<0.001
FFM			
Intercept	4.671 (3.986, 5.356)	13.373	<0.001
Group	1.806 (1.418, 2.194)	9.126	<0.001
Time	−0.394 (−0.621, −0.167)	−3.404	0.001
Group*Time	0.152 (0.021, 0.284)	2.268	0.023

Separate linear mixed-effects models were fitted for the biceps, triceps, subscapular, and suprailiac skinfold thickness, FM, and FFM. The children group (well-nourished and SAM), time (baseline, week 4, week 8, and week 12), and the interaction of the time and group were included as fixed effects. The children were included as random (intercept) effects. SAM: Severe acute malnutrition, FM: Fat-mass, FFM: Fat-free mass.

The rate of increase in the triceps ST was also faster in the children with SAM compared to the well-nourished children (difference in slope = 0.430 mm every 4 weeks; *p* < 0.001). Similarly, the pace of increase in the subscapular (between-group difference in slope = 0.027 mm every 4 weeks; *p* < 0.001) and suprailiac (difference in slope = 0.211 mm every 4 weeks, *p* < 0.001) ST was significantly higher in the SAM group. Similar to regional body fat changes, the overall change in the FM (difference in slope = 0.056 kg every 4 weeks, *p* < 0.001) and FFM (difference in slope = 0.152 kg every 4 weeks, *p* = 0.023) was also significantly faster in the children with SAM compared to the well-nourished children over the 12-week treatment period.

Table 3 shows the changes in the body composition of the boys and girls in the two groups. The girls gained significantly more FM (*β* = 0.20, 95% CI: 0.03, 0.38, *p* = 0.025) and FFM (β = 0.88, 95% CI: 0.13, 1.63, *p* = 0.021) compared to the boys. However, the changes in the FM and FFM of the girls with SAM compared to the well-nourished boys were not significant over the study period.

**Table 3:** Comparative changes in the body composition of the boys and girls of the two groups.

Body composition	β (95% CI)	t	*P*-value
Biceps			
Intercept	3.89 (3.15, 4.63)	10.32	<0.001
Gender	−0.06 (−0.57, 0.43)	−0.23	0.816
Group	0.98 (0.51, 1.46)	4.04	<0.001
Gender*Group	−0.03 (−0.34, 0.28)	−0.20	0.844
Triceps			
Intercept	3.64 (2.70, 4.58)	7.63	<0.001
Gender	0.55 (−0.06, 1.17)	1.76	0.079
Group	2.41 (1.81, 3.01)	7.83	<0.001
Gender*Group	−0.47 (−0.87, −0.09)	−2.43	0.015
Subscapular			
Intercept	3.49 (2.76, 4.21)	9.41	<0.001
Gender	0.28 (−0.20, 0.76)	1.13	0.260
Group	1.77 (1.30, 2.24)	7.42	<0.001
Gender*Group	−0.33 (−0.63, −0.03)	−2.16	0.031
Suprailiac			
Intercept	2.74 (2.02, 3.46)	7.48	<0.001
Gender	1.70 (−0.30, 0.64)	0.70	0.483
Group	1.57 (1.10, 2.03)	6.61	<0.001
Gender*Group	−0.27 (−0.57, 0.03)	−1.77	0.077
FM			
Intercept	0.20 (−0.06, 0.47)	1.50	0.134
Gender	0.20 (0.03, 0.38)	2.25	0.025
Group	0.74 (0.57, 0.91)	8.41	<0.001
Gender*Group	−0.08 (−0.19, 0.03)	−1.40	0.161
FFM			
Intercept	2.36 (1.22, 3.50)	4.06	<0.001
Gender	0.88 (0.13, 1.63)	2.31	0.021
Group	2.53 (1.79, 3.27)	6.72	<0.001
Gender*Group	−0.23 (−0.71, 0.24)	−0.97	0.333

Separate linear mixed-effects models were fitted for the biceps, triceps, subscapular, and suprailiac skinfold thickness, FM, and FFM. The children group (well-nourished and SAM), gender (boys and girls), and the interaction of the gender and group were included as fixed effects. The children were included as random (intercept) effects. SAM: Severe acute malnutrition, FM: Fat-mass, FFM: Fat-free mass.

## 4 Discussion

In Rohingya FDMN camps, children with SAM are treated with RUTF, while the well-nourished children are fed regular foods. RUTF is very high in energy and helps recover and improve weight in the body. The present longitudinal investigation highlighted the changes in the body composition among well-nourished children and children with SAM of 6–59 months in Rohingya refugee camps. The study showed that both groups of children experienced an increase in the overall and regional body fat composition during the 12-week study period. However, the increase in the overall (FM) and regional body (biceps, triceps, subscapular, and suprailiac) composition was faster among the children with SAM compared to the well-nourished children. The change was more pronounced in the first 4 weeks of treatment, and the rate reduced after 4 weeks.

Community Management of Acute Malnutrition (CMAM), recommended by the WHO, is used for the early identification and treatment of children with SAM who do not exhibit medical complications. CMAM includes screening children for SAM, treating medically complicated patients with SAM in-patient, and managing uncomplicated patients with SAM outpatient with RUTF and antibiotics (13). Research suggests that a community-based approach can identify and treat children with SAM well before they develop any health problems (14). Children with SAM are treated with energy-dense RUTF in nutrition emergencies, such as those in Rohingya refugee camps. In the current study, the children with SAM gained both regional and overall FM more rapidly compared to the well-nourished children. FM supplies energy and metabolic precursors for immunological function, which has a significant metabolic cost (15). In addition, FM secretes leptin, which is the immunological system’s “gateway” hormone (16). Low leptin levels have been linked to mortality in children with SAM (17, 18). In recent decades, as more children survive undernutrition in the short term, new concerns about the long-term impacts and implications of treatment have emerged (19, 20). Whether this increase in body fat leads to childhood obesity or obesity in later life needs further investigation. For instance, substantial rates of fat accretion have been reported in previous studies on nutritional treatment for SAM (21, 22). This finding may be partially attributed to diets deficient in micronutrients necessary to facilitate FFM accretion.

In clinical terms, SAM is characterized by a loss of muscle and fat tissue, indicating a diet low in calories and protein compared to requirements. In addition, it has a wider range of pathophysiological problems, such as metabolic dysfunction in cardiac, hepatic, renal, and brain function, all of which are associated with decreased body function (23). Short-term weight gain is commonly used to assess nutritional recovery in young children. However, weight gain during severe illness is primarily due to increases in FM rather than FFM; therefore, an increase in weight does not automatically indicate an increase in FFM. The present study also observed that the children with SAM gained both FM and FFM more rapidly compared to the well-nourished children. Muscle mass can supply essential proteins for immunological function in conditions when there is an insufficient supply of dietary protein or amino acids. Simple indicators of decreased muscle mass are associated with an increased risk of death (24). Although the muscle may be compromised to preserve organs, undernutrition-related thymus shrinkage has been linked to reduced immunological competence (25).

The possibility that the rapid catch-up growth observed during nutritional rehabilitation for SAM is linked to increased body fat deposition and inadequate muscle and visceral protein repletion has been a subject of ongoing debate. However, the current study found no excess fat deposition upon the RUTF supplement in the children with SAM compared with the well-nourished controls. The children who met the anthropometric discharge criteria were nearly identical in fat mass to that of the controls who were well-nourished. The present study found an increased rate of FFM than FM among the children with SAM over the study period. This result is in line with recent findings on the treatment of SAM, where FFM was found to be primarily responsible for weight gain, while RUTF treatment did not increase adiposity (26, 27). Children with mild acute undernutrition in a Cambodian experiment received nutritional supplements for 6–15 months. In this study, there was no change in the fat mass between the four treatment groups that received different RUTF formulations; on average, the fat mass was reduced by 0.2 kg, while the FFM increased by 2.0 kg (28).

According to the current study, during the 12-week investigation, the overall and regional body fat levels increased in both the well-nourished and SAM groups. In contrast to the well-nourished children, the thickness of the biceps, triceps, subscapular, and suprailiac regions increased more rapidly in the children with SAM. This suggests that the children with SAM recovered from their growth faltering faster with the intake of RUTF. During the first 4 weeks of treatment, the change was greater, but after that, the pace decreased in the children with SAM. When the children were discharged from the outpatient therapeutic program, significant differences remained in all the parameters except for the biceps ST between the groups. However, the gap reduced substantially from baseline to discharge. The reduction of the gap indicates quick catch-up growth among the children with SAM. Thus, the findings of the current study corroborate the effectiveness of RUTF in the treatment of these children.

According to the study findings, the girls gained significantly higher triceps ST, subscapular ST, FM, and FFM compared to the boys. As a higher percentage of the participants in the SAM group were girls, they achieved higher levels of FM and FFM upon receiving RUTF, similar to other participants in the same group. However, in the present study, the changes in the FM and FFM among the girls with SAM compared to the well-nourished boys were not significant over time. Previous studies have also shown that boys and girls under 5 years of age exhibit different growth patterns (29–31). However, the gap between the growth patterns is reduced with the increase in the age of the children (31).

The present study has some policy implications. According to our study findings, the content of both FM and FFM was still lower among the cured children with SAM at discharge compared to the well-nourished control groups. Therefore the short-term treatment and discharge criteria should be reevaluated for sustainable recovery and survival from severe and moderate undernutrition. A lower risk of central or peripheral adiposity also ensures the effectiveness of RUTF treatment in children with SAM. However, the development of strategies that integrate rigorous initial nutritional rehabilitation to address muscle deficit and ongoing nutritional support to restore FFM and maintain recovery is necessary. Their efficacy in averting relapse, encouraging linear growth, and facilitating FFM catch-ups could also be evaluated.

The present study has some limitations. It did not collect information on breastfeeding/formula feeds. Therefore, the study could not assess the relationship between breastfeeding/formula feeds and body composition (SFT, FM, and FFM). In addition, the longitudinal associations between body composition and their recovery parameters during rehabilitation were not assessed in the present study. Further studies can explore longitudinal associations between body composition and recovery parameters (anthropometric indices).

## 5 Conclusion

This longitudinal study concludes that the overall and regional body composition of the children with SAM and the well-nourished children increased during the 12-week study period. The rate of increase was significantly rapid in the children with SAM compared to the well-nourished children. The change was higher in the first 4 weeks of treatment. Moreover, the girls gained significantly higher triceps ST, subscapular ST, FM, and FFM compared to the boys. Thus, in this longitudinal study, the benefits of RUTF were evident in the recovery of body weight (FM and FFM) contents among the children with SAM in the Rohingya refugee camps.

## Data Availability

The raw data supporting the conclusions of this article will be made available by the authors, without undue reservation.
