# Supplementary material for: Longitudinal trajectories of the overall and regional body composition between severe acute malnourished and well-nourished children of Rohingya refugee camps
Source: Front Public Health. 2024 Oct 31;12:1442142. doi: 10.3389/fpubh.2024.1442142 (PMC11560770; doi:10.3389/fpubh.2024.1442142)
Supplement: Supplementary file 1 [file Data_Sheet_1.docx]

Supplementary Table 1: Mean difference in body composition between SAM and well-nourished groups at different time points

| Body composition | Well-nourished  Mean | SAM  Mean | Mean difference  (95% CI) | P-value |
| --- | --- | --- | --- | --- |
| Biceps ST (mm) | | | | |
| Admission | 5.51 | 4.21 | 1.29 (1.15, 1.43) | <0.001 |
| Week 4 | 5.62 | 4.72 | 0.89 (0.75, 1.04) | <0.001 |
| Week 8 | 5.73 | 5.23 | 0.50 (0.35, 0.64) | <0.001 |
| Week 12 | 5.84 | 5.74 | 0.10 (-0.04, 0.24) | 0.167 |
| Triceps ST (mm) | | | | |
| Admission | 7.60 | 5.47 | 2.13 (1.94, 2.32) | <0.001 |
| Week 4 | 7.75 | 6.12 | 1.63 (1.45, 1.82) | <0.001 |
| Week 8 | 7.91 | 6.77 | 1.14 (0.95, 1.32) | <0.001 |
| Week 12 | 8.07 | 7.43 | 0.64 (0.45, 0.83) | <0.001 |
| Subscapular ST (mm) | | | | |
| Admission | 6.29 | 1.67 | 1.67 (1.53, 1.81) | <0.001 |
| Week 4 | 6.38 | 1.23 | 1.23 (0.90, 1.38) | <0.001 |
| Week 8 | 6.48 | 0.80 | 0.80 (0.65, 0.94) | <0.001 |
| Week 12 | 6.57 | 0.36 | 0.36 (0.21, 0.50) | <0.001 |
| Suprailiac ST (mm) | | | | |
| Admission | 5.19 | 3.84 | 1.36 (1.21, 1.50) | <0.001 |
| Week 4 | 5.26 | 4.15 | 1.11 (0.97, 1.26) | <0.001 |
| Week 8 | 5.33 | 4.46 | 0.87 (0.73, 1.02) | <0.001 |
| Week 12 | 5.40 | 4.77 | 0.63 (0.49, 0.77) | <0.001 |
| Fat mass (kg) | | | | |
| Admission | 1.69 | 0.98 | 0.71 (0.66, 0.76) | <0.001 |
| Week 4 | 1.73 | 1.11 | 0.62 (0.57, 0.68) | <0.001 |
| Week 8 | 1.77 | 1.24 | 0.53 (0.48, 0.59) | <0.001 |
| Week 12 | 1.81 | 1.36 | 0.44 (0.39, 0.50) | <0.001 |
| Fat-free mass (kg) | | | | |
| Admission | 7.92 | 5.44 | 2.49 (2.25, 2.72) | <0.001 |
| Week 4 | 8.01 | 5.78 | 2.23 (1.99, 2.47) | <0.001 |
| Week 8 | 8.10 | 6.12 | 1.98 (1.74, 2.22) | <0.001 |
| Week 12 | 8.19 | 6.46 | 1.73 (1.49, 1.96) | <0.001 |

SAM: Severe acute malnourished, CI: Confidence interval, ST: Skinfold thickness

P-values were obtained from independent sample t-test

Supplementary Figure 1: Longitudinal trajectories in weight and MUAC among severely acute malnourished (SAM) and well-nourished children; A) Weight, B) MUAC. Predicted values of the body composition parameters were obtained from the linear mixed model.

Supplementary Figure 2: Longitudinal trajectories in body composition among severely acute malnourished (SAM) and well-nourished children; A) Fat mass, B) Fat-free mass. Predicted values of the body composition parameters were obtained from the linear mixed model.
